# Tricarbon­yl(2-methyl-2-η^6^-phenyl-1,3-dioxolane)chromium(0)

**DOI:** 10.1107/S1600536810000759

**Published:** 2010-01-13

**Authors:** Charles B. Duke, Charles R. Ross, Theodore J. Burkey

**Affiliations:** a Department of Pharmaceutical Sciences, University of Tennessee Health Sciences Center, 847 Monroe Avenue, Suite 227A, Memphis, TN 38163, USA; b St. Jude Children’s Research Hospital, Department of Structrual Biology, MS311, 332 North Lauderdale, Memphis, TN 38105-2794, USA; c Department of Chemistry, University of Memphis, 213 Smith Chemistry Building, Memphis, TN 38152-3550, USA

## Abstract

The structure of the title compound, [Cr(C_10_H_12_O_2_)(CO)_3_], is presented. The distorted piano-stool geometry features an off-center Cr(CO)_3_ fragment which reduces contact with the dioxolane ring. The dioxolane ring, in twisted conformation, is *syn*-oriented towards the Cr(CO)_3_ moiety.

## Related literature

For the synthesis of the title compound, see: Bitterwolf (1988 ▶); Mahaffy & Pauson (1990 ▶).
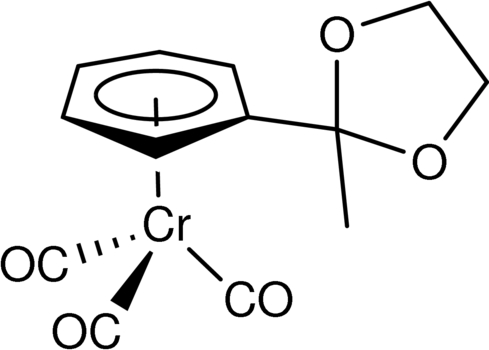


## Experimental

### 

#### Crystal data

[Cr(C_10_H_12_O_2_)(CO)_3_]
*M* *_r_* = 300.23
Triclinic, 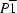

*a* = 7.1950 (3) Å
*b* = 7.2120 (3) Å
*c* = 13.9235 (6) Å
α = 75.573 (2)°
β = 79.277 (2)°
γ = 62.734 (1)°
*V* = 619.79 (5) Å^3^
*Z* = 2
Cu *K*α radiation
μ = 7.74 mm^−1^
*T* = 173 K
0.11 × 0.08 × 0.08 mm

#### Data collection

Bruker Proteum diffractometer
Absorption correction: multi-scan (*SADABS*; Bruker, 2000 ▶) *T*_min_ = 0.489, *T*_max_ = 0.587
13788 measured reflections
2163 independent reflections
2066 reflections with *I* > 2σ(*I*)
*R*_int_ = 0.028

#### Refinement

*R*[*F* ^2^ > 2σ(*F* ^2^)] = 0.028
*wR*(*F* ^2^) = 0.072
*S* = 1.11
2163 reflections
172 parameters
H-atom parameters constrained
Δρ_max_ = 0.34 e Å^−3^
Δρ_min_ = −0.19 e Å^−3^

### 

Data collection: *PROTEUM2* (Bruker, 2005 ▶); cell refinement: *SAINT* (Bruker, 1998 ▶); data reduction: *SAINT*; program(s) used to solve structure: *SHELXS97* (Sheldrick, 2008 ▶); program(s) used to refine structure: *SHELXL97* (Sheldrick, 2008 ▶); molecular graphics: *ORTEP-3* (Farrugia, 1997 ▶) and *RASTER3D* (Merritt & Bacon, 1997 ▶); software used to prepare material for publication: *WinGX* (Farrugia, 1999 ▶).

## Supplementary Material

Crystal structure: contains datablocks global, I. DOI: 10.1107/S1600536810000759/kp2246sup1.cif

Structure factors: contains datablocks I. DOI: 10.1107/S1600536810000759/kp2246Isup2.hkl

Additional supplementary materials:  crystallographic information; 3D view; checkCIF report

## Figures and Tables

**Table:** 

Cr1—C1O	1.837 (2)
Cr1—C3O	1.846 (2)
Cr1—C2O	1.854 (2)
Cr1—C5	2.1942 (18)
Cr1—C6	2.2062 (19)
Cr1—C4	2.2197 (18)
Cr1—C3	2.2248 (18)
Cr1—C2	2.2355 (18)
Cr1—C1	2.2440 (18)
O1C—C1O	1.156 (2)
O3C—C3O	1.150 (2)
O2C—C2O	1.155 (3)

**Table:** 

C1O—Cr1—C3O	85.12 (8)
C1O—Cr1—C2O	90.23 (9)
C3O—Cr1—C2O	88.90 (9)

**Fig. 1.**
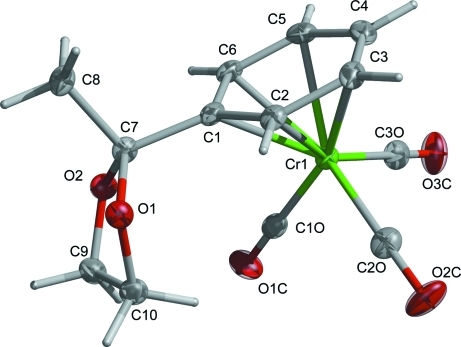
View of I (50% probability displacement ellipsoids) with the dioxolane ring oriented towards the metal center.

**Fig. 2.**
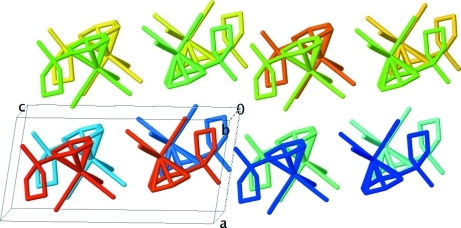
Packing view slightly off of axis b with two unit cells in each dimension.

**Fig. 3.**
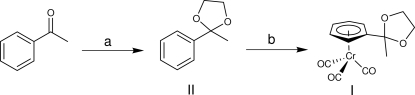
The reaction scheme for the synthesis of I through II Conditions: a) HOCH2CH2OH, PhMe, p-TsOH, 12 h, 25 °C. b) Cr(CO)6, Bu2O/THF (10:1), reflux, 40 h.

## supplementary crystallographic information

### Comment

Synthesized *en route* to η^6^-acetophenone chromium tricarbonyl, I was formed by treatment of acetophenone ethylene ketal (II) with Cr(CO)_6_. Though similar syntheses of the title compound have been previously reported (Bitterwolf, 1981), no structure (Fig. 1) has been previously published. A piano-stool structure, typical of arenechromiumcarbonyls, was found with the sum of the carbonyl C—Cr—C angles of 264.25 (15) °. Rather than above the ring, as would apparently minimize the interaction between the side chain and metal, the dioxolane moiety is oriented towards the Cr(CO)_3_ moiety and the benzylic carbon is approximately 6.5 ° out of the plane of the ring. The distorted piano-stool geometry features an off-center Cr(CO)_3_ fragment which is offset from the dioxolane moiety; Cr—C distances in the ring average 2.2207 (18) Å with a minimum of 2.1942 (18) Å to C5 opposite the closest Cr-sidechain distance of 3.433 Å to H10B. The largest aniosotropic displacement parameters are on the three carbonyl O atoms which experience the largest motion in the molecule as a function of the Cr—CO moment arm. The packing of I (Fig. 2) with two unit cells in each dimension is given.

### Experimental

Acetophenone (30.0 ml, 100 mmol) and ethylene glycol (28.0 ml, 500 mmol) were stirred in toluene (100 ml) with *p*-TsOH (30 mg, 0.17 µmol) for 12 h. Concentration by rotary evaporation and filtration afforded 2 (8.856 g) in 54% yield. The title compound was isolated in 28% yield by the standard literature method (Mahaffey *et al.*, 1990) of treating II with Cr(CO)_6_ in refluxing THF/Bu_2_O (10%) under a nitrogen environment for 40 h. Solvent removal *in vacuo*, filtration and subsequent recrystallization from Et_2_O/hexanes (approximately 1:3 by volume) produced blocky yellow crystals, from which a crystal suitable for diffractometry was selected.

### Refinement

Refinement of all H-atoms was done using isotropic idealized riding models. The largest four peaks in electron density in the model appear in the d-orbitals of chromium, and midway along C9—C10 and C1—C7 bonds.

### Crystal data

**Table:**

[Cr(C_10_H_12_O_2_)(CO)_3_]	*Z* = 2
*M**_r_* = 300.23	*F*(000) = 308
Triclinic, *P*1	*D*_x_ = 1.609 Mg m^−^^3^
Hall symbol: -P 1	Cu *K*α radiation, λ = 1.54178 Å
*a* = 7.1950 (3) Å	Cell parameters from 8653 reflections
*b* = 7.2120 (3) Å	θ = 3.3–68.5°
*c* = 13.9235 (6) Å	µ = 7.74 mm^−^^1^
α = 75.573 (2)°	*T* = 173 K
β = 79.277 (2)°	Block, yellow
γ = 62.734 (1)°	0.11 × 0.08 × 0.08 mm
*V* = 619.79 (5) Å^3^	

### Data collection

**Table:**

Bruker Proteum diffractometer	2163 independent reflections
Radiation source: fine-focus rotating anode	2066 reflections with *I* > 2σ(*I*)
osmic mirrors	*R*_int_ = 0.028
Area detector scans	θ_max_ = 68.7°, θ_min_ = 3.3°
Absorption correction: multi-scan (*SADABS*; Bruker, 2000)	*h* = −8→8
*T*_min_ = 0.489, *T*_max_ = 0.587	*k* = −8→8
13788 measured reflections	*l* = −16→16

### Refinement

**Table:**

Refinement on *F*^2^	0 restraints
Least-squares matrix: full	H-atom parameters constrained
*R*[*F*^2^ > 2σ(*F*^2^)] = 0.028	*w* = 1/[σ^2^(*F*_o_^2^) + (0.0421*P*)^2^ + 0.2689*P*] where *P* = (*F*_o_^2^ + 2*F*_c_^2^)/3
*wR*(*F*^2^) = 0.072	(Δ/σ)_max_ = 0.001
*S* = 1.11	Δρ_max_ = 0.34 e Å^−^^3^
2163 reflections	Δρ_min_ = −0.19 e Å^−^^3^
172 parameters	

### Special details

**Table:**

Geometry. All e.s.d.'s (except the e.s.d. in the dihedral angle between two l.s. planes) are estimated using the full covariance matrix. The cell e.s.d.'s are taken into account individually in the estimation of e.s.d.'s in distances, angles and torsion angles; correlations between e.s.d.'s in cell parameters are only used when they are defined by crystal symmetry. An approximate (isotropic) treatment of cell e.s.d.'s is used for estimating e.s.d.'s involving l.s. planes.

### Fractional atomic coordinates and isotropic or equivalent isotropic displacement parameters (Å^2^)

**Table:**

	*x*	*y*	*z*	*U*_iso_*/*U*_eq_	
Cr1	0.56963 (4)	0.54830 (4)	0.67821 (2)	0.01624 (12)	
O1	0.5396 (2)	0.9647 (2)	0.83565 (10)	0.0215 (3)	
O2	0.64557 (19)	0.6261 (2)	0.92482 (9)	0.0200 (3)	
O1C	0.9718 (2)	0.2980 (2)	0.77613 (11)	0.0285 (3)	
O3C	0.7158 (3)	0.1574 (2)	0.59248 (11)	0.0332 (4)	
O2C	0.7899 (3)	0.7234 (2)	0.50035 (13)	0.0394 (4)	
C7	0.4720 (3)	0.8035 (3)	0.87807 (14)	0.0189 (4)	
C9	0.8237 (3)	0.6734 (3)	0.90280 (15)	0.0223 (4)	
H9A	0.9517	0.5501	0.8844	0.027*	
H9B	0.8483	0.7142	0.9604	0.027*	
C5	0.3036 (3)	0.4871 (3)	0.75700 (15)	0.0212 (4)	
H5	0.2921	0.3565	0.7733	0.025*	
C1	0.4040 (3)	0.7366 (3)	0.79966 (14)	0.0182 (4)	
C2	0.3431 (3)	0.8710 (3)	0.70791 (14)	0.0192 (4)	
H2	0.3577	1	0.6907	0.023*	
C3O	0.6577 (3)	0.3107 (3)	0.62306 (14)	0.0228 (4)	
C10	0.7633 (3)	0.8586 (3)	0.81534 (15)	0.0233 (4)	
H10A	0.8305	0.952	0.8144	0.028*	
H10B	0.8012	0.8088	0.7512	0.028*	
C3	0.2600 (3)	0.8157 (3)	0.64066 (14)	0.0219 (4)	
H3	0.2191	0.9075	0.5786	0.026*	
C6	0.3853 (3)	0.5420 (3)	0.82357 (14)	0.0189 (4)	
H6	0.4285	0.4487	0.885	0.023*	
C1O	0.8162 (3)	0.4006 (3)	0.73882 (14)	0.0203 (4)	
C4	0.2380 (3)	0.6255 (3)	0.66549 (15)	0.0229 (4)	
H4	0.1793	0.5899	0.6211	0.027*	
C2O	0.7044 (3)	0.6586 (3)	0.56913 (16)	0.0256 (4)	
C8	0.2905 (3)	0.8842 (3)	0.95580 (14)	0.0235 (4)	
H8A	0.3349	0.9266	1.0059	0.035*	
H8B	0.2472	0.7712	0.988	0.035*	
H8C	0.1723	1.0068	0.9237	0.035*	

### Atomic displacement parameters (Å^2^)

**Table:**

	*U*^11^	*U*^22^	*U*^33^	*U*^12^	*U*^13^	*U*^23^
Cr1	0.01584 (17)	0.01495 (17)	0.01543 (18)	−0.00518 (12)	−0.00099 (12)	−0.00184 (11)
O1	0.0236 (7)	0.0172 (6)	0.0221 (7)	−0.0082 (5)	−0.0042 (6)	−0.0003 (5)
O2	0.0184 (6)	0.0184 (6)	0.0191 (7)	−0.0061 (5)	−0.0045 (5)	0.0017 (5)
O1C	0.0184 (7)	0.0246 (7)	0.0405 (9)	−0.0037 (6)	−0.0096 (6)	−0.0085 (6)
O3C	0.0481 (9)	0.0220 (7)	0.0257 (8)	−0.0089 (7)	−0.0069 (7)	−0.0080 (6)
O2C	0.0429 (9)	0.0283 (8)	0.0347 (9)	−0.0147 (7)	0.0155 (8)	−0.0002 (7)
C7	0.0196 (9)	0.0157 (9)	0.0176 (9)	−0.0056 (7)	−0.0026 (8)	−0.0001 (7)
C9	0.0208 (9)	0.0233 (10)	0.0234 (10)	−0.0097 (8)	−0.0048 (8)	−0.0030 (8)
C5	0.0156 (8)	0.0211 (9)	0.0259 (11)	−0.0088 (7)	0.0025 (8)	−0.0040 (8)
C1	0.0132 (8)	0.0191 (9)	0.0161 (9)	−0.0028 (7)	0.0013 (7)	−0.0033 (7)
C2	0.0164 (8)	0.0148 (8)	0.0200 (10)	−0.0021 (7)	−0.0001 (7)	−0.0029 (7)
C3O	0.0249 (10)	0.0231 (10)	0.0173 (10)	−0.0090 (8)	−0.0048 (8)	0.0012 (8)
C10	0.0231 (9)	0.0238 (10)	0.0236 (10)	−0.0116 (8)	−0.0018 (8)	−0.0025 (8)
C3	0.0171 (9)	0.0219 (9)	0.0188 (10)	−0.0019 (8)	−0.0047 (8)	−0.0016 (7)
C6	0.0156 (8)	0.0203 (9)	0.0161 (9)	−0.0063 (7)	0.0016 (7)	−0.0001 (7)
C1O	0.0222 (10)	0.0194 (9)	0.0209 (10)	−0.0107 (8)	0.0038 (8)	−0.0073 (7)
C4	0.0150 (8)	0.0278 (10)	0.0252 (10)	−0.0067 (8)	−0.0037 (8)	−0.0075 (8)
C2O	0.0252 (10)	0.0170 (9)	0.0274 (12)	−0.0040 (8)	−0.0005 (9)	−0.0036 (8)
C8	0.0241 (9)	0.0220 (10)	0.0188 (10)	−0.0053 (8)	−0.0013 (8)	−0.0038 (7)

### Geometric parameters (Å, °)

**Table:**

Cr1—C1O	1.837 (2)	C9—H9A	0.99
Cr1—C3O	1.846 (2)	C9—H9B	0.99
Cr1—C2O	1.854 (2)	C5—C6	1.401 (3)
Cr1—C5	2.1942 (18)	C5—C4	1.415 (3)
Cr1—C6	2.2062 (19)	C5—H5	0.95
Cr1—C4	2.2197 (18)	C1—C2	1.402 (3)
Cr1—C3	2.2248 (18)	C1—C6	1.422 (3)
Cr1—C2	2.2355 (18)	C2—C3	1.417 (3)
Cr1—C1	2.2440 (18)	C2—H2	0.95
O1—C7	1.416 (2)	C10—H10A	0.99
O1—C10	1.437 (2)	C10—H10B	0.99
O2—C7	1.430 (2)	C3—C4	1.403 (3)
O2—C9	1.434 (2)	C3—H3	0.95
O1C—C1O	1.156 (2)	C6—H6	0.95
O3C—C3O	1.150 (2)	C4—H4	0.95
O2C—C2O	1.155 (3)	C8—H8A	0.98
C7—C8	1.519 (3)	C8—H8B	0.98
C7—C1	1.530 (3)	C8—H8C	0.98
C9—C10	1.520 (3)		
			
C1O—Cr1—C3O	85.12 (8)	C6—C5—Cr1	71.91 (10)
C1O—Cr1—C2O	90.23 (9)	C4—C5—Cr1	72.29 (11)
C3O—Cr1—C2O	88.90 (9)	C6—C5—H5	119.9
C1O—Cr1—C5	115.71 (8)	C4—C5—H5	119.9
C3O—Cr1—C5	88.65 (8)	Cr1—C5—H5	128.1
C2O—Cr1—C5	153.62 (8)	C2—C1—C6	119.20 (17)
C1O—Cr1—C6	91.04 (8)	C2—C1—C7	121.36 (16)
C3O—Cr1—C6	114.92 (8)	C6—C1—C7	119.23 (16)
C2O—Cr1—C6	156.17 (8)	C2—C1—Cr1	71.43 (10)
C5—Cr1—C6	37.12 (7)	C6—C1—Cr1	69.92 (10)
C1O—Cr1—C4	152.90 (8)	C7—C1—Cr1	135.38 (12)
C3O—Cr1—C4	90.08 (8)	C1—C2—C3	120.40 (17)
C2O—Cr1—C4	116.37 (8)	C1—C2—Cr1	72.09 (10)
C5—Cr1—C4	37.38 (7)	C3—C2—Cr1	71.06 (10)
C6—Cr1—C4	66.92 (7)	C1—C2—H2	119.8
C1O—Cr1—C3	157.39 (8)	C3—C2—H2	119.8
C3O—Cr1—C3	117.47 (8)	Cr1—C2—H2	129.5
C2O—Cr1—C3	91.19 (8)	O3C—C3O—Cr1	177.08 (17)
C5—Cr1—C3	66.90 (7)	O1—C10—C9	101.71 (15)
C6—Cr1—C3	78.82 (7)	O1—C10—H10A	111.4
C4—Cr1—C3	36.80 (7)	C9—C10—H10A	111.4
C1O—Cr1—C2	120.35 (8)	O1—C10—H10B	111.4
C3O—Cr1—C2	154.47 (8)	C9—C10—H10B	111.4
C2O—Cr1—C2	92.49 (8)	H10A—C10—H10B	109.3
C5—Cr1—C2	78.90 (7)	C4—C3—C2	120.15 (17)
C6—Cr1—C2	66.52 (7)	C4—C3—Cr1	71.40 (10)
C4—Cr1—C2	66.54 (7)	C2—C3—Cr1	71.89 (10)
C3—Cr1—C2	37.05 (7)	C4—C3—H3	119.9
C1O—Cr1—C1	93.34 (7)	C2—C3—H3	119.9
C3O—Cr1—C1	152.18 (8)	Cr1—C3—H3	129.1
C2O—Cr1—C1	118.90 (8)	C5—C6—C1	120.38 (17)
C5—Cr1—C1	66.98 (7)	C5—C6—Cr1	70.98 (11)
C6—Cr1—C1	37.27 (7)	C1—C6—Cr1	72.81 (11)
C4—Cr1—C1	78.72 (7)	C5—C6—H6	119.8
C3—Cr1—C1	66.38 (7)	C1—C6—H6	119.8
C2—Cr1—C1	36.48 (7)	Cr1—C6—H6	128.7
C7—O1—C10	106.41 (13)	O1C—C1O—Cr1	176.41 (16)
C7—O2—C9	108.32 (13)	C3—C4—C5	119.66 (18)
O1—C7—O2	106.85 (14)	C3—C4—Cr1	71.80 (11)
O1—C7—C8	108.85 (15)	C5—C4—Cr1	70.34 (10)
O2—C7—C8	109.64 (15)	C3—C4—H4	120.2
O1—C7—C1	112.00 (15)	C5—C4—H4	120.2
O2—C7—C1	109.66 (14)	Cr1—C4—H4	130.2
C8—C7—C1	109.78 (15)	O2C—C2O—Cr1	178.51 (18)
O2—C9—C10	103.74 (14)	C7—C8—H8A	109.5
O2—C9—H9A	111	C7—C8—H8B	109.5
C10—C9—H9A	111	H8A—C8—H8B	109.5
O2—C9—H9B	111	C7—C8—H8C	109.5
C10—C9—H9B	111	H8A—C8—H8C	109.5
H9A—C9—H9B	109	H8B—C8—H8C	109.5
C6—C5—C4	120.18 (17)		
